# The microdialysis-derived lactate–pyruvate gradient indicates anaerobic activity after brain injury

**DOI:** 10.1177/0271678X261485404

**Published:** 2026-08-26

**Authors:** Michael S Baker, Joshua M Heihre, Tomasz Kuliński, Claudia A Smith, Berfin Barlas, Chisomo Zimphango, Ivan Timofeev, Koby Baranes, Keri L H Carpenter, Mark Kotter, Mathew R Guilfoyle, Elham Rostami, Peter J Hutchinson, Adel Helmy

**Affiliations:** 1 Department of Clinical Neurosciences, University of Cambridge, Cambridge, UK; 2 Section of Neurosurgery, Department of Medical Sciences, Uppsala University Hospital, Uppsala, Sweden; 3 Wellcome-MRC Cambridge Stem Cell Institute, University of Cambridge, Cambridge, UK; 4 Department of Physiology and Pharmacology, Karolinska Institutet, Stockholm, Sweden

**Keywords:** Cerebral metabolism, changepoint detection, mitochondrial dysfunction, neuromonitoring, neurocritical care

## Abstract

Cerebral microdialysis (CMD) after traumatic brain injury (TBI) has focused on lactate/pyruvate ratio (LPR), overlooking lactate–pyruvate gradient (LPG, the linear regression slope within a time segment of CMD datapoints) as a proxy for anaerobic activity. We retrospectively applied changepoint detection to the linear lactate–pyruvate relationship (L = *m*P + *b*) over time within patients from two independent CMD-monitored TBI cohorts (Cambridge, *n* = 510; Uppsala, *n* = 81), estimating per-segment LPG (*m*) and intercept (*b*, capturing metabolic variance). In vitro, we tested whether rotenone-induced mitochondrial dysfunction increases LPG in hiPSC-derived neurons, as expected if LPG tracks anaerobic activity. In the Cambridge cohort, a median of two linear segments were identified per patient (median LPG = 19.83; median *b* = 0.54 mM). Between segments, LPR tracked LPG with heavy attenuation (β = 0.255). LPG was negatively associated with cerebral microdialysate glucose (*p* < 0.001) and 6-month GOSE (*p* = 0.039). Uppsala data confirmed pipeline robustness and replicated the LPG–glucose association (*p* = 0.022). LPG was higher in rotenone-treated neurons than controls (32.9 vs 14.2, *p* < 0.001). LPG calculated per time segment provides a more accurate, outcome-associated proxy for anaerobic activity than datapoint-level LPR.

## Introduction

**Figure 1.**
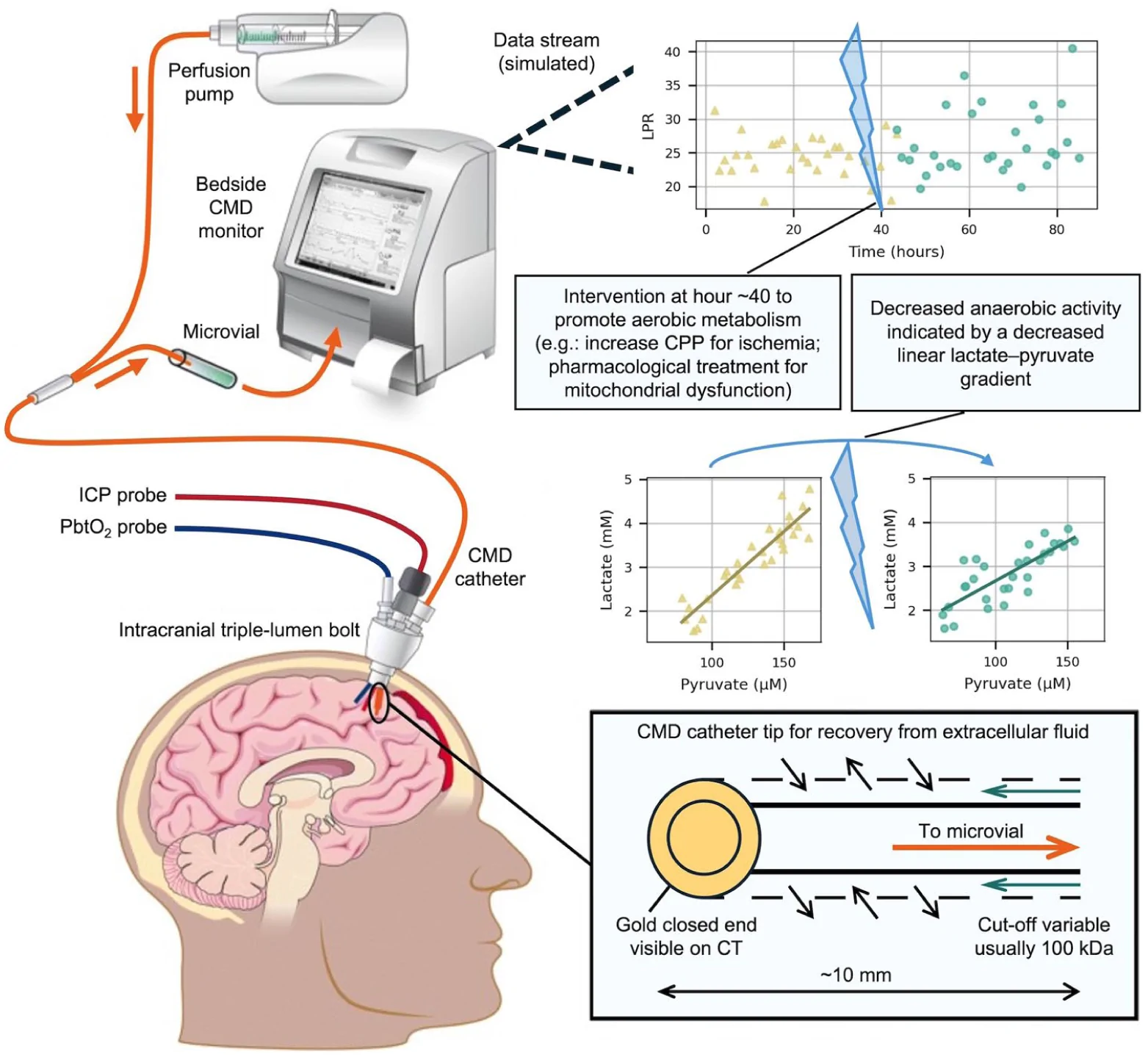
Cerebral microdialysis and the linear lactate–pyruvate framework. *Left*: schematic of the intracranial multimodality monitoring bolt and CMD catheter (ICP and PbtO_2_ probes shown). *Bottom right*: close-up of the CMD catheter tip sampling extracellular fluid. CMD schematic adapted from Khellaf et al.^9^ under Creative Commons License (CC BY). *Top–middle right*: simulated CMD data illustrating the proposed framework, generated using representative parameters (cohort medians) from the Cambridge dataset. *Top right*: LPR versus time for two contiguous segments over ~83 h, with a hypothetical intervention at hour ~40. Despite the underlying lactate–pyruvate gradient (LPG) decreasing from 29.04 to 17.97 between segments, the observed LPR remains in a similar, overlapping range, illustrating how the conventional LPR time series can obscure true changes in anaerobic activity. *Middle right*: corresponding lactate versus pyruvate scatter plots with OLS regression lines. The LPG of the first segment (gold triangles; Type N_*b*_, LPG = 29.04, *b* = −0.55 mM) reflects higher anaerobic activity than the second segment (teal circles; Type P_*b*_, LPG = 17.97, *b* = 0.87 mM). *b*: linear intercept; CMD: cerebral microdialysis; ICP: intracranial pressure; LPG: lactate–pyruvate gradient; LPR: lactate/pyruvate ratio; N_*b*_: negative intercept (*b* < 0); OLS: ordinary least squares; P_*b*_: positive intercept (*b* > 0); PbtO_2_: brain tissue partial pressure of oxygen.

Cerebral metabolic derangement is a detrimental secondary injury mechanism following traumatic brain injury (TBI).^1^ Cerebral microdialysis (CMD) is an invasive technique used in neurocritical care in which a probe placed into the brain parenchyma samples extracellular fluid, enabling monitoring of local biochemistry and metabolic derangements (Figure 1). The lactate/pyruvate ratio (LPR) derived from CMD has thus far served as the key clinical indicator of metabolic distress and anaerobic activity.^2^ An elevated cerebral LPR is associated with poorer neurological^3–6^ and brain tissue^7,8^ outcomes.

However, the traditional interpretation of CMD data relies on isolated LPR measurements assessed against fixed thresholds (e.g. 25),^3,10–13^ collapsing the interaction between lactate and pyruvate into a single value that obscures their linear co-variation. Here, we introduce a framework for characterising the linear lactate–pyruvate relationship to infer anaerobic activity more accurately than via datapoint-level LPR. We use ‘anaerobic activity’ to denote an LDH-linked shift in cytosolic redox state associated with impaired oxidative metabolism (by reduced oxygen availability or delivery, or mitochondrial dysfunction), not complete tissue anoxia.

Throughout this manuscript, we use three distinct terms. (i) The lactate/pyruvate ratio (LPR) is the standard datapoint-level, unitless CMD metric of lactate divided by pyruvate at a single timepoint. (ii) The linear lactate–pyruvate relationship is estimated by fitting lactate against pyruvate (L = *m*P + *b*) within a time segment of CMD datapoints. (iii) We term the unitless slope *m* of this linear relationship the lactate–pyruvate gradient (LPG); intercept *b* absorbs metabolic variance. We hypothesise that changes in LPG between segments track shifts in anaerobic activity more accurately than unadjusted, datapoint-level LPR fluctuations.

Our analyses were inspired by our past studies,^13,14^ which identified a notable polarisation in the metabolic response to injury among TBI patients. These studies revealed a U-shaped distribution in the prevalence of LPR > 25, with one substantial group of patients never demonstrating this metabolic derangement and another always demonstrating LPR > 25. We anticipated this polarisation may manifest from differences in linear lactate–pyruvate relationships. Building on our group’s earlier linear modelling of the lactate–pyruvate relationship,^15^ with the lactate dehydrogenase (LDH) enzyme’s linkage of the two substrates serving as the theoretical basis, we hypothesised that this linear relationship changes over time within patients, with distinct segments characterised by different LPG and *b*.

We pursue several interconnected objectives:

1. Develop an automated changepoint detection pipeline to retrospectively identify shifts in the CMD-derived linear lactate–pyruvate relationship between data segments within each patient.
2. Validate that segment-level linear fits outperform patient-level fits and that the intercept term *b* is empirically necessary.
3. Characterise LPG (*m*) and *b*, including their within-patient variation.
4. Quantify how LPR is confounded by *b* due to the hyperbolic LPR–pyruvate relationship (equation (6)).
5. Explain the previously observed polarisation in LPR > 25 prevalence by linking it to fundamental differences in linear lactate–pyruvate relationships.^13,14^
6. Assess how faithfully LPR tracks LPG and test whether LPG is associated with substrate delivery, as indicated by cerebral microdialysate glucose.
7. Test whether LPG is associated with functional outcome (6-month GOSE).
8. Assess the generalisability of our findings in an independent TBI cohort.
9. Test whether rotenone-induced mitochondrial dysfunction increases LPG in an in vitro neuronal model, providing mechanistic evidence that LPG reflects anaerobic activity.

## Materials and methods

### Study population

This retrospective analysis used a Cambridge source cohort of 619 acute TBI patients aged ⩾16 years (median age 37 years, IQR 24–52; 76.3% male), monitored at Addenbrooke’s Hospital, Cambridge, UK, from December 1997 to January 2017. Clinical management and multimodal monitoring are detailed in Supplemental Materials SM1; prior cohort analyses have been published.^3,6,13^

The independent Uppsala cohort comprised 81 CMD-monitored severe TBI patients (median age 39 years, IQR 20–56; 75.3% male) admitted to Uppsala University Hospital, Sweden, from 2008 to 2020; cohort and management details are in SM3.

### Data integration and pre-processing

The present study draws on the integrated, pre-processed multimodal dataset that our group generated for earlier studies.^3,6^ The raw data, 56,307 observations across 619 patients, were imported using custom R scripts. The resulting dataset, comprising 56,250 multimodal observations from 616 patients, was accessed on May 1, 2025, after anonymisation. The dataset was then subjected to additional pre-processing for linear lactate–pyruvate dynamics. The pre-processing pipeline produced a cleaned dataset containing 50,797 total observations (with complete data for lactate, pyruvate, and LPR) across 510 patients. In the analysed Cambridge and Uppsala cohorts, CMD lactate and pyruvate were sampled at approximately hourly intervals; the CMD data included in the analysis spanned a median of 5.2 days per patient (Cambridge IQR 3.4–8.0; Uppsala IQR 3.4–7.4; Segmentation pipeline output, Supplemental Tables S1 and S4). Full pre-processing details may be found in SM2 for the Cambridge cohort and SM3.iii for the Uppsala cohort.

### Linear characterisation of lactate–pyruvate data

#### Theoretical basis

The intracellular LDH reaction is the foundation of our framework centred on the linear lactate–pyruvate relationship. This reaction is fast and reversible, operating in near-equilibrium within the living cell:

(1) $$Lactate+NAD^{+}\overset{LDH}{↔}Pyruvate+NADH+H^{+}$$

At near-equilibrium, the enzyme maintains the products/reactants ratio (the mass-action ratio) very close to the reaction’s thermodynamic equilibrium constant (K_eq_). Thus, the relationship is described by the law of mass action:

(2) $$K_{eq}\approx \frac{\left[Pyruvate\right]\left[NADH\right]\left[H^{+}\right]}{\left[Lactate\right]\left[NAD^{+}\right]}$$

We can rearrange this equation to define the relationship between the CMD-measured metabolites, lactate (L) and pyruvate (P):

(3) $$L\approx \left(\frac{1}{K_{eq}}\times \left[H^{+}\right]\times \frac{\left[NADH\right]}{\left[NAD^{+}\right]}\right)\times P$$

The entire parenthetical term in equation (3) is a composite parameter that we denote *m* and refer to as the lactate–pyruvate gradient (LPG):

(4) L=mP

LPG (*m*) remains stable for a segment of data when the product $\left(\frac{\left[NADH\right]}{\left[NAD^{+}\right]}\times [H^{+}]\right)$ is stable; this does not require that the redox state or proton concentration be individually constant. Equation (4) represents a simplified model relative to equation (5). An empirical model with intercept *b* can be fit to account for remaining metabolic factors, such as those arising from non-LDH-linked substrate sources or sinks:

(5) L=mP+b

Thus, the intercept term *b* can account for some non-LDH-linked contributions to lactate and pyruvate, enabling LPG (*m*) to more selectively reflect the LDH-linked redox signal.

Crucially, when *b* ≠ 0, dividing equation (5) by pyruvate reveals a hyperbolic relationship between LPR and pyruvate (see equation (6)) in which LPR is displaced from LPG (*m*) and varies with substrate concentration even when LPG reflects a stable underlying metabolic signal.

(6) $$LPR=\frac{L}{P}=m+\frac{b}{P}$$

Supplemental Figure S1 illustrates this transformation conceptually, demonstrating how the sign and magnitude of *b* determine the direction and degree of curvature of the resulting LPR–pyruvate hyperbola, while the LPG (*m)* sets the horizontal asymptote toward which the LPR converges at high pyruvate concentrations.

Our framework operates on two assumptions: that the LDH reaction operates near equilibrium,^16^ and that extracellular CMD measurements retain information about aggregate local intracellular LDH-linked redox dynamics due to monocarboxylate transporters (MCT1, MCT2, and MCT4) on neuronal and glial cells facilitating a degree of intracellular–extracellular metabolic coupling.^17^ We therefore use LPG to infer changes in anaerobic activity between data segments over time.

#### Segmentation pipeline

To identify temporally distinct linear segments within each patient’s lactate–pyruvate data (equation (5)), we developed an automated segmentation pipeline using changepoint detection. The pipeline employs the Pruned Exact Linear Time (PELT) algorithm^18,19^ to detect changepoints in the linear lactate–pyruvate relationship over time, with a minimum segment length of six datapoints. The optimal number of segments was then selected using the Kneedle algorithm^20,21^ to identify the elbow point on the cost-complexity curve. Segments partition each patient’s CMD data series into temporally ordered, non-overlapping blocks of datapoints separated at boundaries by one sampling interval (~1 h). Throughout, ‘segment’ denotes such a time segment of CMD datapoints. Changepoints are introduced when the data indicate a metabolic transition large enough to warrant a fitted shift in the linear lactate–pyruvate relationship, rather than pinpointing the instantaneous time of metabolic change. Ordinary least squares (OLS) regression was then fit within each resulting segment to obtain segment-level LPG (*m*) and intercept (*b*) values. For downstream analyses, we retained only segments with positive LPG (*m* > 0), consistent with *m* > 0 as implied by equation (3), where *m* comprises only positive terms under near-equilibrium LDH kinetics. Complete pipeline details and code are provided in the published repository.

### In vitro model

To independently test whether LPG is sensitive to anaerobic activity, we induced mitochondrial dysfunction in a reductionist in vitro hiPSC-derived neuronal model. Glutamatergic neurons were generated via optimised inducible overexpression (opti-ox) of neurogenin 2 (NGN2) in hiPSCs and maintained at 37°C in a humidified 5% CO_2_ atmosphere (full protocol in SM4). On Day 21 post-induction, neurons underwent a complete media change to a test medium supplemented with 5 mM glucose without exogenous lactate, pyruvate, or L-glutamine, containing either rotenone (10 nM, ⩾95% purity; Sigma-Aldrich, St. Louis, MO, USA; a complex I inhibitor of the mitochondrial electron transport chain [ETC], dissolved in DMSO) or vehicle control. Samples were collected from parallel wells at timepoints between 1 and 24 h post-treatment and analysed on ISCUSflex analysers (M Dialysis AB, Stockholm, Sweden), mirroring our clinical workflow.

### Statistical analysis and code availability

Multiple statistical approaches were used, including OLS regression, Fisher’s z-transformed correlation comparisons, model comparison via Akaike Information Criterion (AIC), linear mixed-effects (LME) models with nested random effects, nonparametric tests for group comparisons with Benjamini-Hochberg correction for multiple testing, linear contrasts via estimated marginal means (EMMs), and analysis of covariance (ANCOVA) for comparing in vitro regression lines. EMMs represent the model-estimated typical values within each group, adjusted for the model’s random effects and weighting structure. All analyses were performed using R (v4.5.2; https://www.r-project.org/) and Python (v3.9.6; https://www.python.org/). The complete, annotated source code of this project is publicly available at https://github.com/msb-jr/LPG. The relation of each script to each section of the manuscript and Supplementary materials is specified in the repository. Where directional hypotheses were pre-specified based on theoretical expectation or established literature (e.g. higher LPR associated with worse outcomes), one-sided tests were used; all other tests were two-sided. The directionality of each test is specified in the published code. The Uppsala cohort was analysed using the same published pipeline and scripts; all statistical methods were identical to those applied to the Cambridge cohort.

### Ethical considerations

This study was conducted in accordance with the Declaration of Helsinki. For the Cambridge cohort, the head injury research programme was approved by the Cambridge Local Research Ethics Committee (REC reference: 97/290; IRAS project ID: 39184) on December 5, 1997. Addenbrooke’s Hospital’s Research and Development Department also approved the study. For the Uppsala cohort, ethical approval was granted by the Uppsala County branch of the Swedish Ethical Review Authority (approval numbers: #2010/138, #2010/138/1, #2010/379, #2015/224, and #2020-05462). The in vitro component used human induced pluripotent stem cell-derived neurons and did not involve human participants; separate ethical approval was not required.

### Consent to participate

For the Cambridge cohort, informed consent was obtained from the next of kin or a legally authorised representative, in written or verbal form, with provision for deferred consent. For the Uppsala cohort, written informed consent was obtained from next-of-kin.

## Results

The following Results sections describe the Cambridge cohort unless otherwise specified.

### Segmentation pipeline output

The lactate–pyruvate linear segmentation pipeline was applied to 50,797 datapoints across 510 patients (median 88 datapoints per patient, IQR 56–135, spanning a median of 5.2 days, IQR 3.4–8.0 days). The pipeline identified 1465 data segments in total. After retaining only segments with positive LPG (*m* > 0), 1339 segments (91.4%) comprising 48,504 datapoints (95.5%) were retained across 506 patients (99.2%).

**Figure 2.**
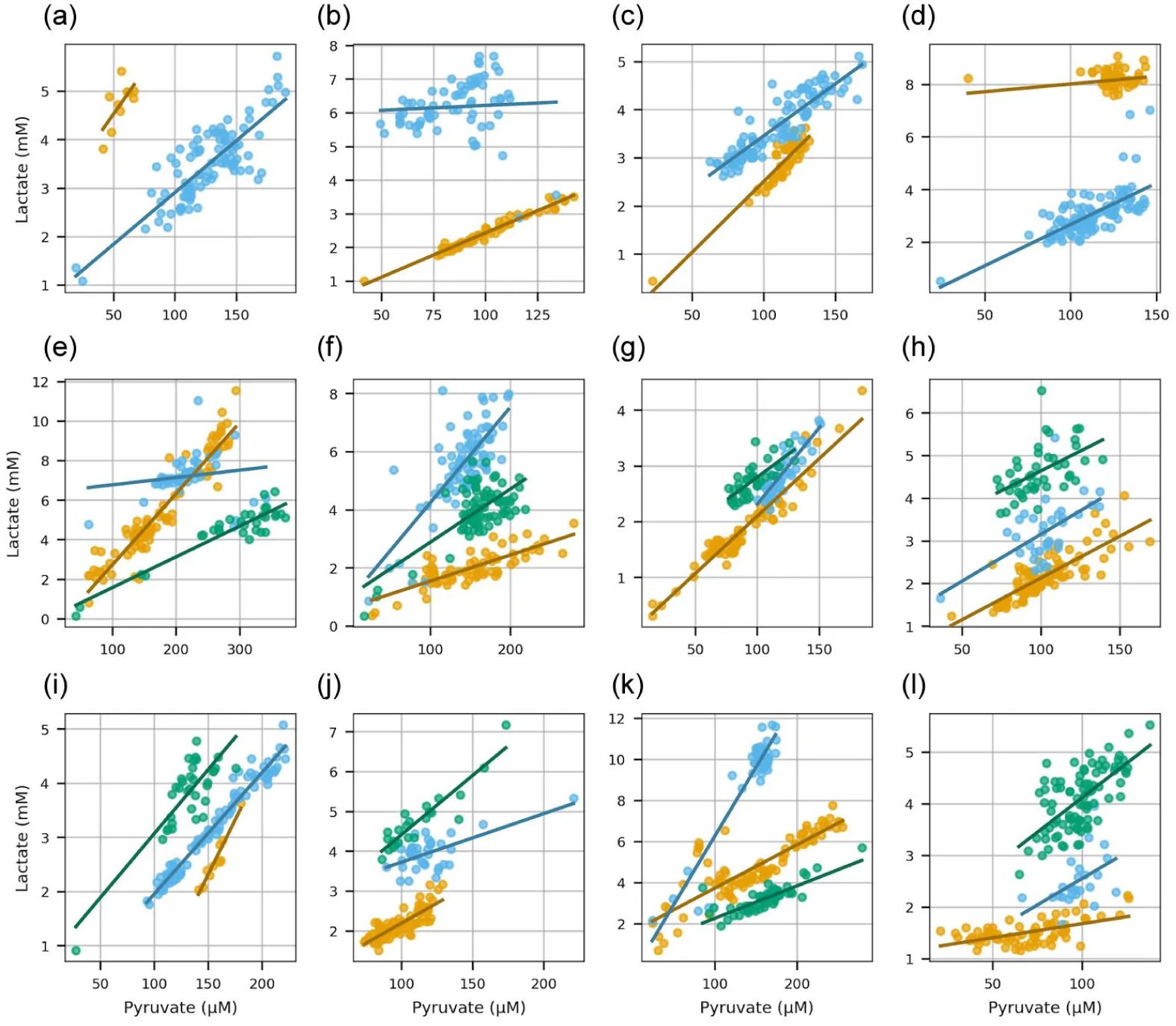
Lactate–pyruvate segmentation results for 12 representative patients. Each panel (a–l) shows one patient’s segment-level scatter plots with ordinary least squares regression lines. Datapoints are coloured according to their assigned segment, which are temporally distinct and sequential; for any given patient, all datapoints belonging to ‘Segment 1’ occur before those in ‘Segment 2’, and so on. Each segment comprises a contiguous block of hourly observations bounded by changepoints. Segments are distinctly coloured in temporal order (orange first, blue second, then green). All segments shown for these patients had positive lactate–pyruvate gradients (*m* > 0).

Retained segments contained a median of 30 datapoints (IQR 20–42) collected over a median span of 41.4 h (IQR 28.3–60.4 h). Each retained patient had a median of two retained segments (IQR 2–3; range 1–7). Segmentation details are reported in Supplemental Table S1. Figure 2 visualises segmentation results for representative patients. Supplemental Figure S2 illustrates the complete pipeline process for a patient in whom one segment was discarded due to a non-positive LPG.

### Validation of the linear framework

We validated two core assumptions of our framework. First, we tested whether fitting multiple linear segments per patient captures the lactate–pyruvate relationship more accurately than fitting a single regression line to each patient’s entire monitoring period. A weighted LME model on Fisher’s z-transformed Pearson correlations (1465 segments across 510 patients) confirmed that segment-level correlations (EMM *r* = 0.83, 95% CI: 0.82–0.85) were significantly higher than patient-level correlations (EMM *r* = 0.75, 95% CI: 0.73–0.77; *p* < 0.001), indicating that the positive linear lactate–pyruvate relationship is more clearly resolved when patients’ data is segmented and justifying the use of changepoint-based segmentation. All subsequent analyses were performed exclusively on retained segments (*m* > 0).

Second, we tested whether including a non-zero intercept (*b*) improves segment-level linear fits compared to an origin-constrained model (L = *m*P, equation (4)). Across 1339 retained segments from 506 patients, the typical improvement was substantial (ΔAIC = 10.45, 95% CI: 9.46–11.45; *p* < 0.001), with 57.2% of individual segments showing a clear preference for the unconstrained model (ΔAIC > 2). These results empirically confirm that the two-parameter model (L = *m*P + *b*, equation (5)) better describes the observed lactate–pyruvate data, supporting the inclusion of the intercept to isolate the LPG as a proxy for anaerobic activity.

### Linear parameters and the hyperbolic consequence

#### Characterisation

**Figure 3.**
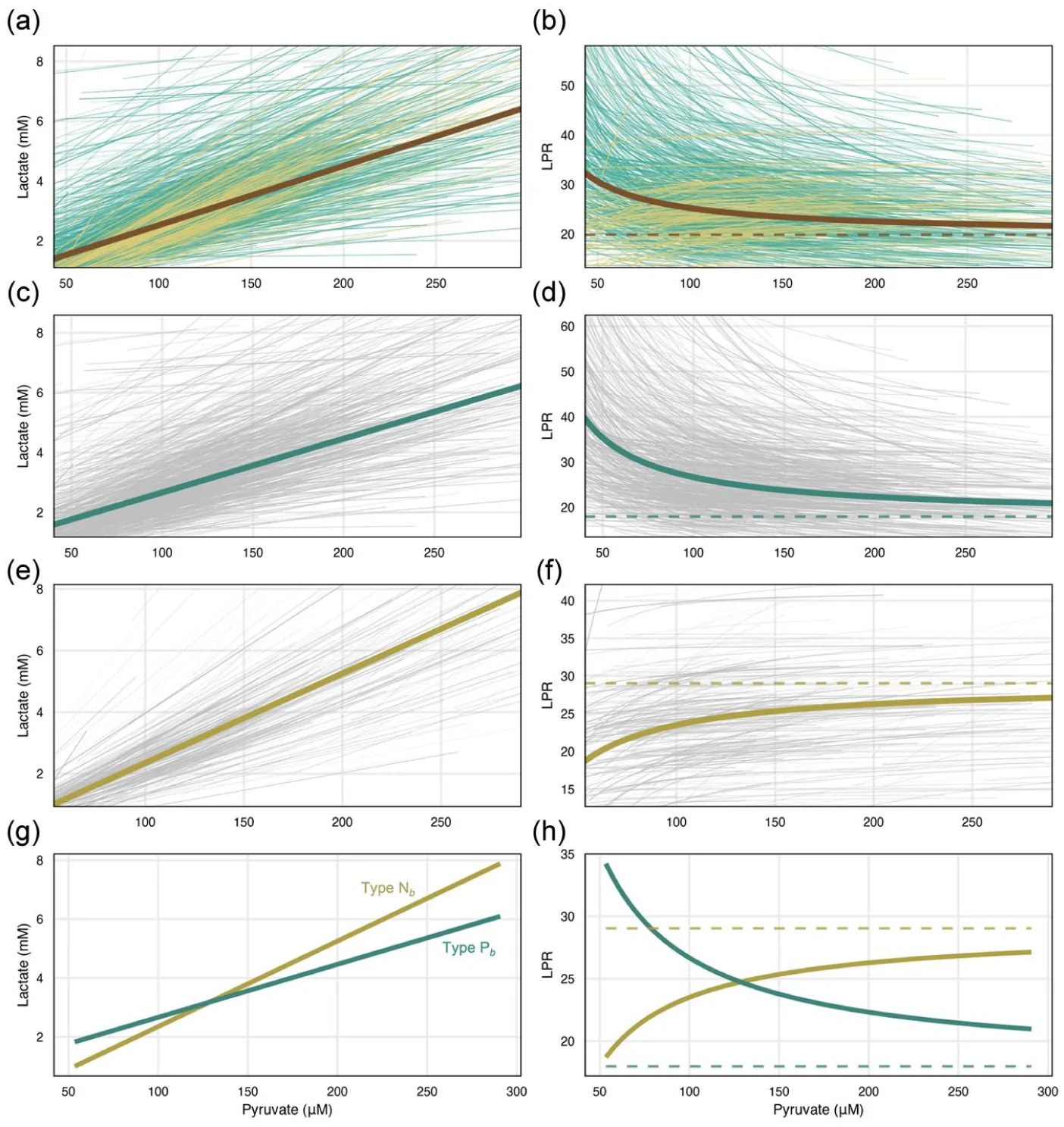
Empirical lactate–pyruvate relationships across retained segments. Left panels display lactate versus pyruvate (linear space); right panels display LPR versus pyruvate (hyperbolic space). Thin lines represent individual segment-level ordinary least squares fits; thick lines represent the cohort median trend. Dashed horizontal lines indicate the LPG (*m*) asymptote. (a–b) Unstratified: all 1339 retained segments from 506 patients, coloured by intercept type (teal = Type P_*b*_, gold = Type N_*b*_) with cohort median trend (brown). (c–d) Type P_*b*_ segments: 967 segments, 476 patients. Positive intercepts produce LPR hyperbolae that decrease toward the LPG asymptote as pyruvate increases. (e–f) Type N_*b*_ segments: 372 segments, 276 patients. Negative intercepts produce LPR hyperbolae that increase toward the LPG asymptote as pyruvate increases. (g–h) Combined overlay comparing Type P_*b*_ and Type N_*b*_ cohort median trends. *b*: linear intercept; LPG (*m*): lactate–pyruvate gradient; LPR: lactate/pyruvate ratio; N_*b*_: negative intercept (*b* < 0); P_*b*_: positive intercept (*b* > 0).

Across the 1339 retained segments of 506 patients (Figure 3(a) and (b)), the cohort median LPG was 19.83 (IQR: 15.48–25.10) and the median intercept (*b*) was 0.54 mM (IQR: 0.13–1.12 mM), with a median within-segment *R*^2^ of 0.69 (IQR: 0.54–0.80). Among the 466 patients with two or more retained segments, within-patient variation was substantial: the median intra-patient range of LPG was 12.28 (IQR: 6.33–21.20) and for *b* was 1.69 mM (IQR: 0.77–3.00 mM).

Stratification by the sign of the intercept revealed two distinct patterns (Figure 3(c)–(h)). Segments with a positive intercept (Type P_*b*_; 967 [72.2%] segments, 476 patients; median *b* = 0.87 mM; Figure 3(c) and (d)) had lower LPG (median LPG = 17.97, IQR: 13.33–22.03) and moderate linear fits (median *R*^2^ = 0.63, IQR: 0.48–0.77), producing decreasing LPR hyperbolae as pyruvate increased (Figure 3(d)). Segments with a negative intercept (Type N_*b*_; 372 [27.8%] segments, 276 patients; median *b* = −0.55 mM; Figure 3(e) and (f)) had markedly higher LPG (median LPG = 29.04, IQR: 23.83–35.72) and stronger linear fits (median *R*^2^ = 0.85, IQR: 0.74–0.91), producing increasing LPR hyperbolae toward the asymptote equal to the LPG (*m*) (Figure 3(f)). These empirical patterns mirror the conceptual framework illustrated in Supplemental Figure S1.

#### Within-segment LPR error

**Figure 4.**
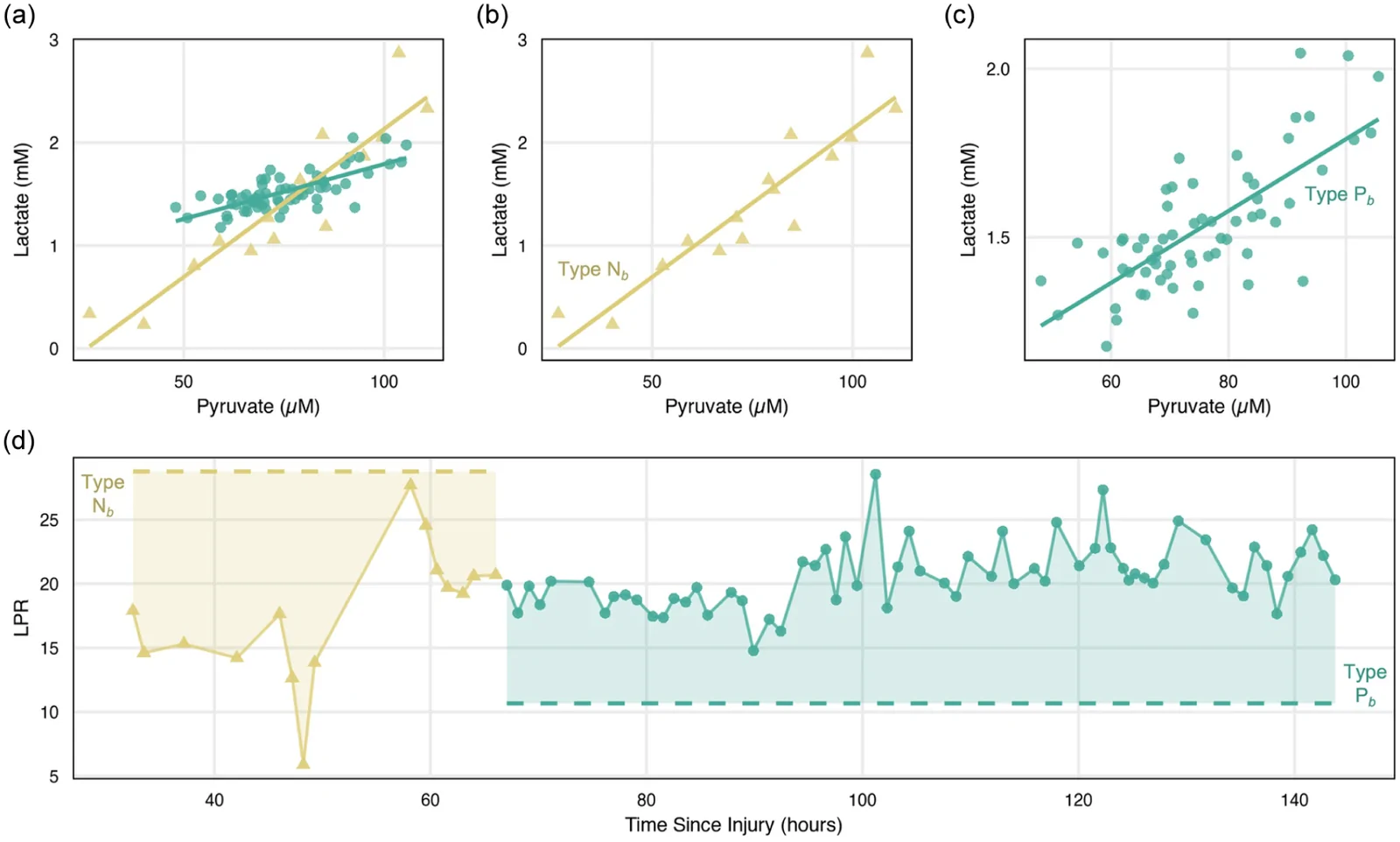
LPR deviation from LPG in a representative patient. (a) lactate versus pyruvate for the patient’s two retained segments (linear fits overlaid), (b) the initial Type N_*b*_ segment (gold triangles; LPG = 28.76, *b* = −0.74 mM, *r* = 0.92), (c) the subsequent Type P_*b*_ segment (teal circles; LPG = 10.67, *b* = 0.72 mM, *r* = 0.74), and (d) LPR (y-axis) plotted against time since injury (hours) for both segments. Dashed horizontal lines indicate the LPG for each segment; shaded regions highlight the deviation of the observed LPR from LPG. In the Type N_*b*_ segment, LPR (range: 5.9–27.7) consistently underestimated the LPG, while in the Type P_*b*_ segment, LPR (range: 14.8–28.5) consistently exceeded the LPG. Despite the LPG decreasing from 28.76 to 10.67 between segments, the observed LPR remained in a similar range due to the opposing effects of the intercept, illustrating how LPR can obscure true changes in anaerobic activity. *b*: linear intercept; LPG: lactate–pyruvate gradient; LPR: lactate/pyruvate ratio; N_*b*_: negative intercept (*b* < 0); P_*b*_: positive intercept (*b* > 0); *r*: Pearson correlation coefficient.

The presence of a non-zero intercept causes the LPR to deviate from the underlying LPG in a substrate-dependent manner (Supplemental Table S2). This deviation is most pronounced at low pyruvate concentrations: at the segment-minimum pyruvate (median 64 µM, IQR: 41–89 µM), the overall median relative LPR error was 66.8% (IQR: 20.2%–190.6%). In Type P_*b*_ segments, the LPR exceeded the LPG by a median of 103.4% (IQR: 39.1%–254.7%) at minimum pyruvate, while in Type N_*b*_ segments, the LPR underestimated the LPG by a median of −19.9% (IQR: −38.0% to −8.6%). Even at higher substrate levels (segment-maximum pyruvate, median 172 µM, IQR: 139–225 µM), the median LPR error remained 23.3% (IQR: 7.1%–55.6%). Furthermore, LPR varied substantially within segments despite a stable LPG: the median within-segment LPR range was 14.4 (IQR: 9.2–27.6), driven entirely by substrate-dependent fluctuations along the hyperbolic curve. These findings demonstrate that the LPR systematically fails to track the level of anaerobic activity reflected by the LPG, with the direction and magnitude of the deviation determined by the sign and magnitude of the intercept *b* and the prevailing pyruvate concentration. Figure 4 demonstrates this mismatch in one patient’s time series, where the LPR remained in a similar range despite a near-threefold decrease in the LPG between segments.

### Explaining metabolic polarisation

To explain the previously observed polarisation of LPR values around the clinical threshold of 25,^13,14^ we compared the linear parameters of two cohorts: patients whose LPR always exceeded 25 (‘Always LPR > 25’; 39 patients, 82 retained segments) and those whose LPR never exceeded 25 (‘Never LPR > 25’; 38 patients, 92 retained segments). Notably, both cohorts had similarly negligible proportions of datapoints in discarded segments (*m* ⩽ 0) prior to filtering (median 0.0% in both), supporting the comparability of the two groups on the basis of retained data.

The ‘Always LPR > 25’ cohort had significantly higher LPG (median LPG = 27.39, IQR: 23.82–33.78) compared to the ‘Never LPR > 25’ cohort (median LPG = 14.59, IQR: 12.44–18.20; *p* < 0.001), significantly higher intercepts (median *b* = 1.62 mM, IQR: 0.58–2.76 vs 0.19 mM, IQR: −0.15–0.37; *p* < 0.001), and a significantly higher proportion of Type P_*b*_ segments (median 100.0% vs 66.3%; *p* < 0.001) (Supplemental Table S3). These differences in underlying linear parameters produce fundamentally different hyperbolic LPR–pyruvate curves: the combination of higher LPG and higher positive *b* in the ‘Always’ cohort generates LPR values that are generally above 25 across the physiological pyruvate range, while the lower LPG and near-zero *b* in the ‘Never’ cohort produce flatter or inverted curves that keep LPR below the threshold. Thus, the previously unexplained polarisation of LPR about 25 is a direct mathematical consequence of differences in the underlying linear lactate–pyruvate relationships.

### Substrate delivery and LPR fidelity

**Figure 5.**
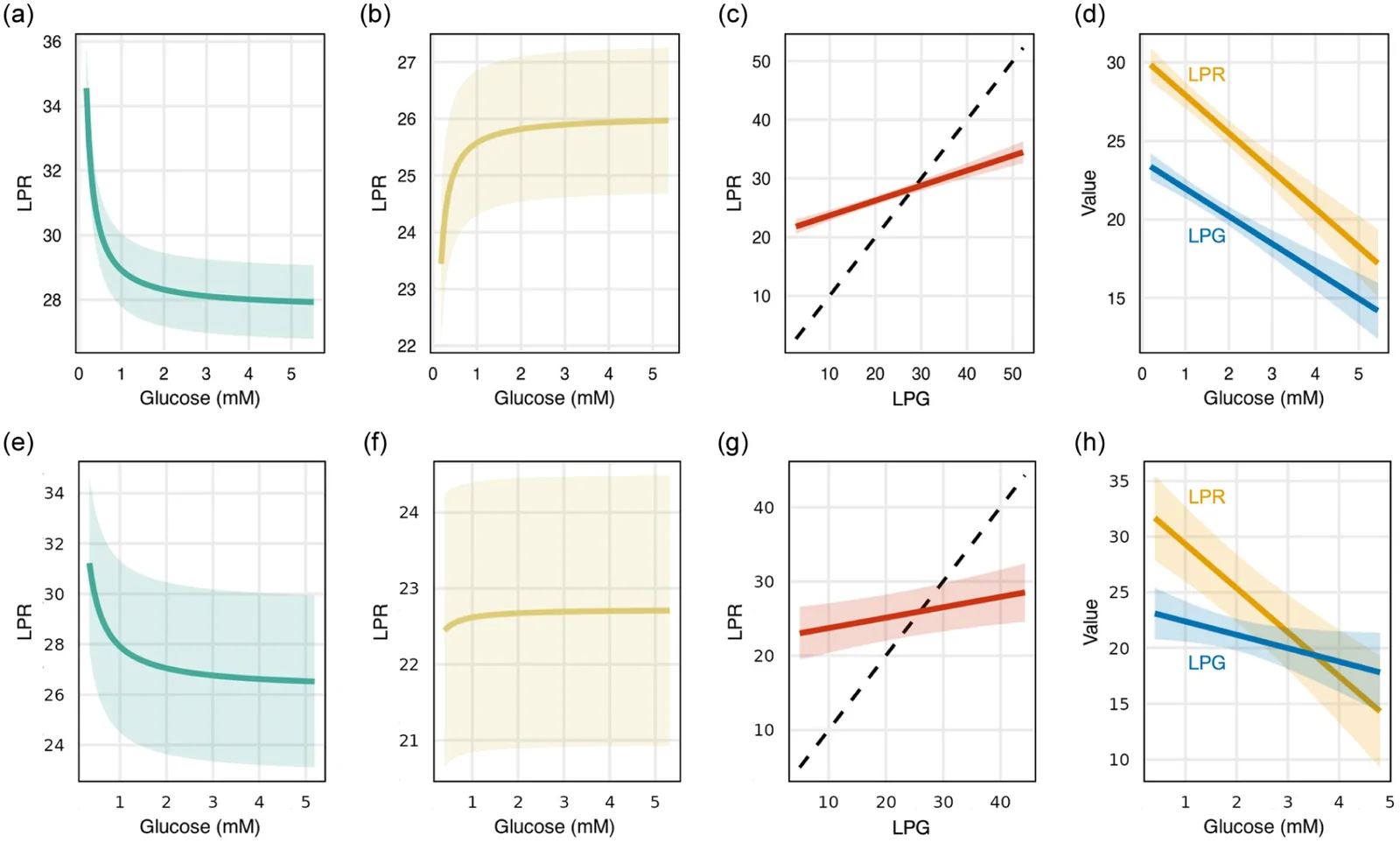
Substrate delivery and LPR fidelity. (a) Within-segment LPR versus glucose for Type P_*b*_ segments (954 segments, 474 patients): LPR decreases with increasing glucose (β_1/glucose_ = 1.21 mM, *p* < 0.001). (b) Within-segment LPR versus glucose for Type N_*b*_ segments (367 segments, 272 patients): LPR increases with increasing glucose (β_1/glucose_ = −0.48 mM, *p* < 0.001). (a–b) Within-segment models use 1/Glucose as predictor to capture the hyperbolic LPR–glucose relationship (LPR = *m* + *b*/P); predictions are plotted on the glucose axis; nested random intercepts (segment within patient); *p*-values are Benjamini-Hochberg corrected (*n* = 2). (c) Segment-level median LPR versus LPG (β = 0.255, *p* < 0.001); dashed black line indicates the theoretical β = 1 relationship expected if LPR perfectly tracked LPG. (d) Segment-level LPG (blue; β = −1.75 mM^−1^, *p* < 0.001) and median LPR (orange; β = −2.41 mM^−1^, *p* < 0.001) versus median glucose. (c–d) Patient random intercepts, weighted by segment size. (a–d) Cambridge cohort; lines represent LME fixed effects; shaded regions represent 95% CIs; sample: 1321 segments, 504 patients. (e–h) Corresponding analyses for the Uppsala cohort (215 segments, 81 patients). (e) Type P_*b*_ (132 segments, 72 patients; β_1/glucose_ = 1.72 mM, *p* < 0.001). (f) Type N_*b*_ (83 segments, 61 patients; β_1/glucose_ = −0.11 mM, *p* = 0.090). (g) Median LPR versus LPG (β = 0.140, *p* = 0.005). (h) LPG versus glucose (β = −1.20 mM^−1^, *p* = 0.022) and median LPR versus glucose (β = −3.95 mM^−1^, *p* < 0.001). *b*: linear intercept; CI: confidence interval; LME: linear mixed-effects model; LPG (*m*): lactate–pyruvate gradient; LPR: lactate/pyruvate ratio; N_*b*_: negative intercept (*b* < 0); P_*b*_: positive intercept (*b* > 0).

Next, we investigated the relationships between cerebral glucose (a proxy for substrate delivery), LPR, and the linear parameters LPG (*m*) and *b* (Figure 5(a)–(d)). Glucose has a dual association with the system: it tracks segment-level anaerobic activity (LPG), and it simultaneously drives pyruvate supply, generating artefactual LPR fluctuations through the hyperbolic relationship (equation (6)). At the level of individual observations, glucose was strongly and positively associated with pyruvate (β = 13.71 µM/mM, *p* < 0.001; residual SD = 30.8 µM), supporting that glucose drives pyruvate and validating the examination of hyperbolic LPR–glucose associations.

Within segments of stable LPG, the LPR exhibited a hyperbolic association with glucose that depended on the sign of the intercept *b* (Figure 5(a) and (b)). In Type P_*b*_ segments, increasing glucose was associated with decreasing LPR (*p* < 0.001; Figure 5(a)), while in Type N_*b*_ segments the association reversed (*p* < 0.001; Figure 5(b); *p*-values Benjamini-Hochberg corrected, *n* = 2).

At the between-segment level, the LPR correlated positively with LPG (*p* < 0.001; Figure 5(c)), but the association was heavily attenuated (β = 0.255, i.e. 0.255-unit increase in segment median LPR per unit increase in LPG, compared to a theoretical β = 1 if LPR perfectly tracked LPG). Part of the attenuation is explained by a significant negative coupling between LPG and *b* (β = −0.097 mM per unit LPG, *p* < 0.001). Given equation (6) (LPR = *m* + *b*/P): when LPG (*m*) increases and *b* simultaneously decreases, the opposing change in *b* partially offsets the effect of higher LPG on the LPR.

Finally, the LPG showed a robust negative association with segment-level median glucose (β = −1.75 mM^−1^, *p* < 0.001; Figure 5(d)), consistent with the expected physiological response: reduced substrate delivery is associated with increased anaerobic activity. The segment-level median LPR was also negatively associated with glucose (β = −2.41 mM^−1^, *p* < 0.001; Figure 5(d)), but with a coefficient larger in magnitude than that of LPG. This disproportionately large response is consistent with the summation of two effects: (i) the true physiological signal (lower LPG at higher glucose) and (ii) a Type P_*b*_ (72.2% of segments) substrate-dependent artefact (higher glucose → higher pyruvate → deflated ^+^*b*/P term → further LPR deflation). Thus, while both LPG and LPR decrease with increasing glucose, LPR’s response conflates the anaerobic signal with the intercept-dependent artefact, whereas LPG isolates the physiological relationship.

### Outcome association

Additionally, we tested whether the linear parameters LPG (*m*) and *b* are associated with 6-month functional outcome using the Glasgow Outcome Scale-Extended (GOSE), summarised into four categories: Death (GOSE 1; 87 patients, 217 segments), Severe disability (GOSE 2–4; 117 patients, 310 segments), Moderate disability (GOSE 5–6; 160 patients, 423 segments), and Good recovery (GOSE 7–8; 112 patients, 305 segments).

**Figure 6.**
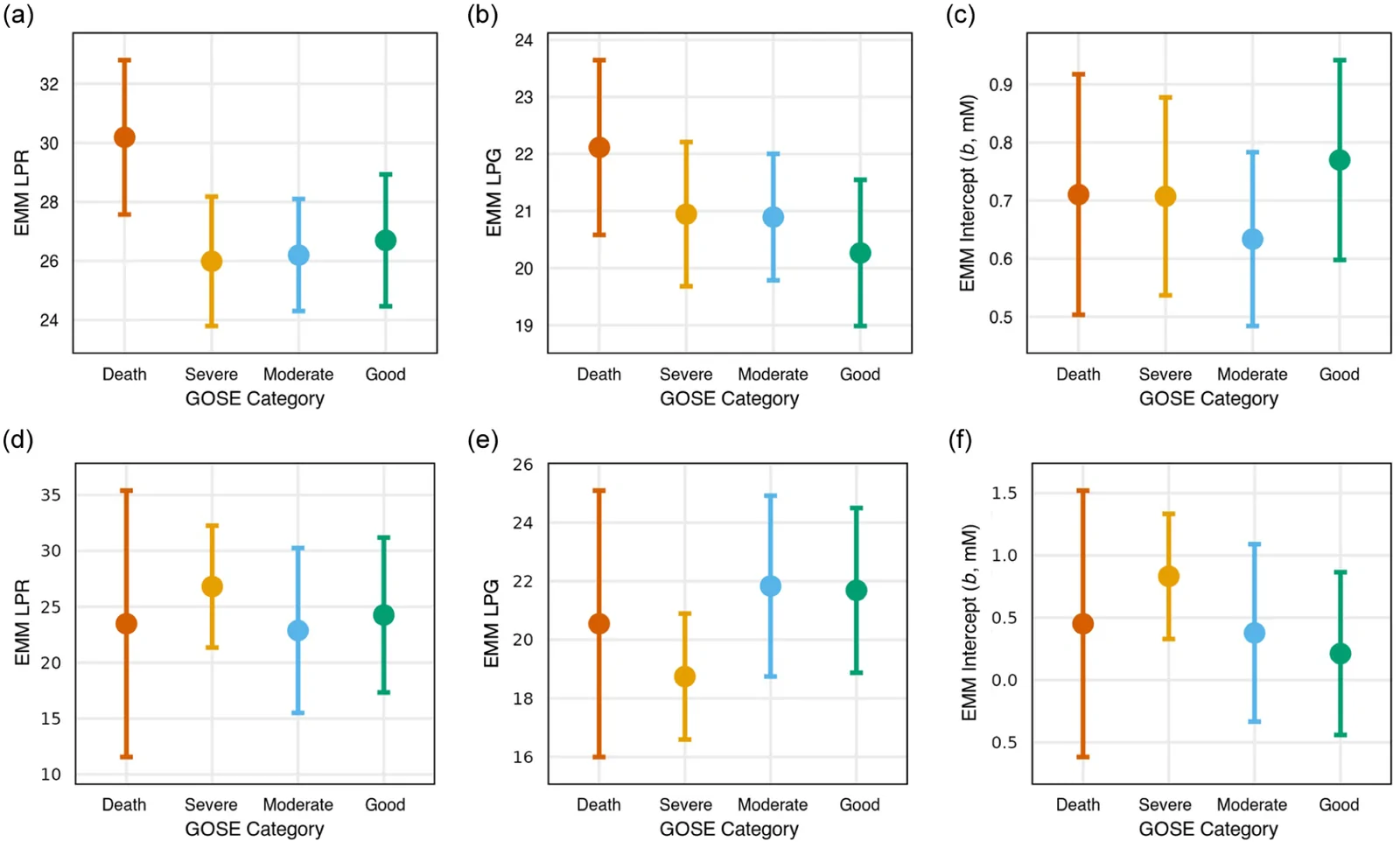
Estimated marginal means of linear lactate–pyruvate parameters by 6-month functional outcome. (a) segment median LPR, (b) segment LPG, and (c) segment *b* across four GOSE categories: Death (GOSE 1; 87 patients, 217 segments), Severe disability (GOSE 2–4; 117 patients, 310 segments), Moderate disability (GOSE 5–6; 160 patients, 423 segments), and Good recovery (GOSE 7–8; 112 patients, 305 segments). Points represent EMMs from weighted LME models (weights = segment size) with patient random intercepts; error bars represent 95% CIs. Linear contrasts tested for monotonic trends across outcome categories: (a) LPR, β = −10.27 (*p* = 0.030); (b) LPG, β = −5.59 (*p* = 0.039); (c) *b*, β = 0.10 (*p* = 0.806). (a–c) Cambridge cohort. (d–f) Corresponding analyses for the Uppsala cohort: Death (6 patients, 20 segments), Severe (29 patients, 74 segments), Moderate (16 patients, 41 segments), Good (18 patients, 52 segments). Linear contrasts: (d) LPR, β = −1.59 (*p* = 0.470); (e) LPG, β = 6.51 (*p* = 0.784); (f) *b*, β = −1.17 (*p* = 0.546). *b*: linear intercept; CI: confidence interval; EMM: estimated marginal mean; GOSE: Glasgow Outcome Scale-Extended; LME: linear mixed-effects model; LPG: lactate–pyruvate gradient; LPR: lactate/pyruvate ratio.

The EMMs for each outcome group are shown in Figure 6(a)–(c). Validating established literature,^3,11^ LPR was significantly associated with outcome (linear contrast *p* = 0.030; Figure 6(a)). However, the LPR–outcome trend was largely driven by the Death group (EMM = 30.2, 95% CI: 27.6–32.8), with the Severe, Moderate, and Good groups clustering closely together (EMMs: 26.0, 26.2, 26.7).

Crucially, higher values of LPG were also significantly associated with worse outcome (linear contrast *p* = 0.039; Figure 6(b)), but unlike the LPR, the LPG showed a consistent monotonic trend across all four outcome categories, decreasing from 22.1 (95% CI: 20.6–23.6) in the Death group to 20.3 (95% CI: 19.0–21.5) in the Good recovery group. This graded relationship supports the clinical relevance of LPG: segments belonging to patients with worse neurological outcomes were characterised by a steeper linear lactate–pyruvate relationship (higher LPG), indicating higher levels of anaerobic cerebral metabolism.

The intercept *b* showed no significant association with outcome (linear contrast *p* = 0.806; Figure 6(c)), consistent with its role as an empirical adjustment parameter rather than a direct indicator of the LDH-linked metabolic signal.

### Independent Uppsala cohort

To assess generalisability, an independent centre (Uppsala University Hospital, Uppsala, Sweden) applied the segmentation pipeline and all downstream analyses to a TBI cohort of 81 patients (8150 CMD observations; SM3). The pipeline identified 232 segments, of which 215 (92.7%) were retained (*m* > 0), encompassing 7781 datapoints (95.5%) across all 81 patients (100%). Each patient had a median of two retained segments (IQR 2–3; Supplemental Table S4 and Figure S3).

Segment-level Pearson correlations (EMM *r* = 0.83) were significantly higher than patient-level correlations (EMM *r* = 0.80; *p* = 0.015), replicating the Cambridge finding and confirming that segmentation improves the resolution of the linear lactate–pyruvate relationship. The intercept model (L = *m*P + *b*) significantly outperformed the origin-constrained model (typical ΔAIC = 7.00, 95% CI: 4.85–9.15, *p* < 0.001), with 42.8% of segments showing a clear preference for the unconstrained model (ΔAIC > 2).

Across the retained segments, the median LPG (*m*) was 20.16 (IQR: 17.16–24.93), median intercept (*b*) was 0.12 mM (IQR: −0.15 to 0.76), and median within-segment *R*^2^ was 0.66 (IQR: 0.53–0.78). Among the 76 patients with two or more retained segments, the median intra-patient range of LPG was 11.36 (IQR: 5.91–18.23). LPR varied substantially within segments despite stable LPG (median within-segment LPR range: 10.4, IQR: 7.4–14.3; Supplemental Table S5).

The within-segment hyperbolic LPR–glucose association was significant in Type P_*b*_ segments (*p* < 0.001; Figure 5(e)) but did not reach significance in Type N_*b*_ segments (*p* = 0.090; Figure 5(f)). Glucose was positively associated with pyruvate (β = 16.38 µM/mM, *p* < 0.001; residual SD = 28.3 µM). The segment-level association between LPR and LPG (β = 0.140, *p* = 0.005; Figure 5(g)) replicated the direction of the Cambridge finding (β = 0.255), with both falling substantially short of the theoretical β = 1 if LPR perfectly tracked LPG. The heavier attenuation of the LPR–LPG association in the Uppsala cohort is in line with the more negative coefficient of the LPG–*b* relationship (β = −0.142 mM per unit LPG, *p* < 0.001). The LPG was negatively associated with segment-level median glucose (β = −1.20 mM^−1^, *p* = 0.022), while segment-level median LPR demonstrated a more extreme trend with glucose (β = −3.95 mM^−1^, *p* < 0.001; Figure 5(h)) consistent with amplification from an additional substrate-dependent artefact associated with the majority of segments’ Type P_*b*_ identity (61.4%).

In the Uppsala cohort (69 patients with outcome data: Death, 6; Severe, 29; Moderate, 16; Good, 18), neither LPR (linear contrast *p* = 0.470) nor the LPG (linear contrast *p* = 0.784) were significantly associated with 6-month GOSE. The intercept *b* also showed no significant association (linear contrast *p* = 0.546; Figure 6(d)–(f)).

### In vitro validation

**Figure 7.**
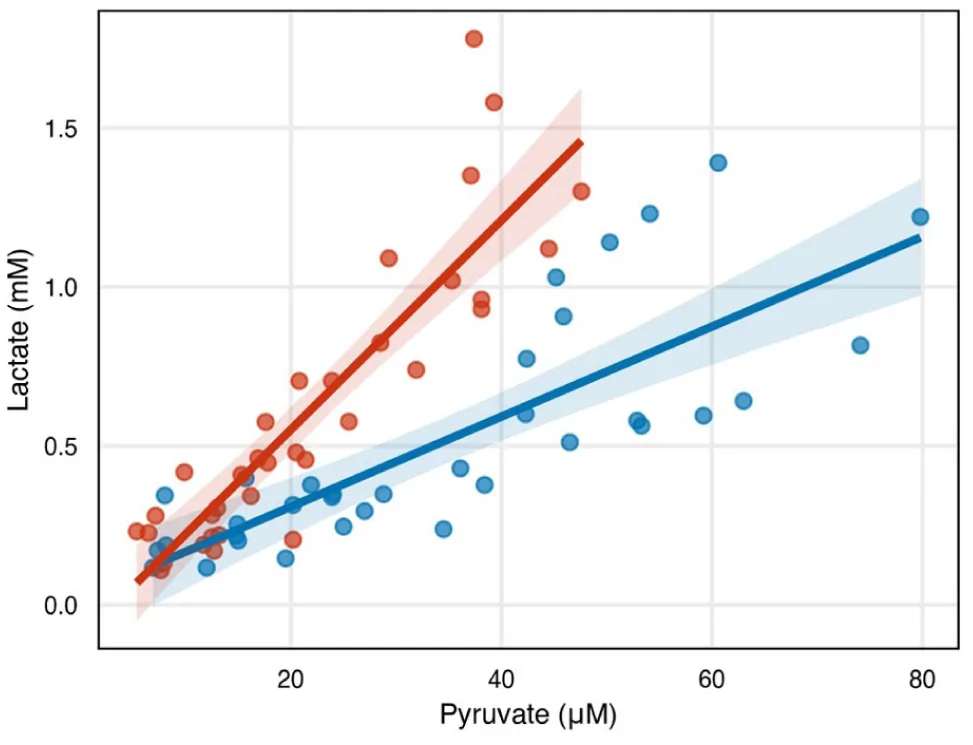
In vitro lactate–pyruvate relationship under rotenone-induced mitochondrial dysfunction. Lactate versus pyruvate for control (blue; *n* = 34; LPG = 14.2, *b* = 0.026 mM, *R*^2^ = 0.66) and rotenone-treated (red; *n* = 34; LPG = 32.9, *b* = −0.107 mM, *R*^2^ = 0.80) hiPSC-derived neurons. ANCOVA confirmed a significant shift in LPG (*p* < 0.001) and *b* (*p* < 0.001; residual df = 64). Points represent individual measurements from four independent experiments, each condition having ⩾2 technical replicates, pooled across timepoints between 1 and 24 h. Lines represent OLS regression fits; shaded regions represent 95% CIs. ANCOVA: analysis of covariance; *b*: linear intercept; CI: confidence interval; hiPSC: human induced pluripotent stem cell; LPG: lactate–pyruvate gradient; OLS: ordinary least squares.

To test whether LPG (*m)* is sensitive to anaerobic activity, we induced mitochondrial dysfunction in hiPSC-derived neurons with rotenone (10 nM), a complex I inhibitor, and examined the linear lactate–pyruvate relationship (Figure 7). Under control conditions, neurons exhibited an LPG of 14.2 (*R*^2^ = 0.66, *n* = 34). Rotenone treatment more than doubled LPG to 32.9 (*R*^2^ = 0.80, *n* = 34), with the difference confirmed by ANCOVA (LPG shift *p* < 0.001; residual df = 64). The intercept (*b*) was near zero in both conditions (control: 0.026 mM; rotenone: −0.107 mM), though the shift was also significant (*p* < 0.001). These results provide direct evidence that inhibition of the mitochondrial ETC increases the LPG, consistent with the interpretation of LPG as an indicator of anaerobic activity.

## Discussion

This study introduces a framework for isolating the lactate–pyruvate gradient (LPG, *m*) estimated by linear regression (equation (5), L = *m*P + *b*) as a more direct and stable indicator of anaerobic activity than the LPR. To our knowledge, this is the first study to systematically characterise the linear lactate–pyruvate relationship as monitored by CMD and to track it over time within individual patients. We demonstrate that the LPG is negatively associated with substrate delivery, significantly associated with functional outcome, and mechanistically linked to mitochondrial dysfunction, and we replicated core empirical findings in the Uppsala cohort. In contrast, the LPR, although also associated with outcome, is systematically displaced from the LPG by the linear intercept (*b*), which captures non-LDH-linked metabolic variance. Thus, the LPR is a less reliable marker of anaerobic activity.

The hypothesis that LDH-driven lactate–pyruvate dynamics are detectable via CMD and linear modelling was strongly supported by our data: 91.4% of detected linear segments had positive LPG, consistent with *m* (equations (3) and (4)) comprising only positive terms under near-equilibrium LDH kinetics. These retained segments had a median duration of 41.4 h and a median goodness of fit of *R*^2^ = 0.69. The minority of segments with non-positive LPG (8.6%) may reflect Simpson’s paradox^22^ arising from insufficient temporal resolution, or biological complexity such as non-LDH-linked lactate sources (e.g. cell death) (Supplemental Figure S2). The high retention rate indicates that the linear lactate–pyruvate relationship is a robust and prevalent feature of CMD data in TBI.

A key methodological contribution of this study was the use of data-driven, automated changepoint detection to identify temporally distinct linear trends within each patient’s monitoring data. This approach was empirically validated: segment-level correlations significantly exceeded patient-level correlations, confirming that patients experience multiple distinct linear relationships throughout their monitoring rather than a single static trend. The substantial within-patient variation in LPG (median range 12.28) further underscores that the level of anaerobic activity is not fixed but shifts over time, potentially reflecting changes in substrate delivery, mitochondrial function, or therapeutic intervention.

The empirical necessity of the intercept (*b*) was confirmed by AIC model comparison. While *b* is essential for accurately isolating LPG, its presence destabilises the LPR: given equation (6) (LPR = *m* + *b*/P), LPR deviates from LPG (*m*) in a substrate-dependent manner amplified at low pyruvate. This error was striking: in positive-intercept segments, median LPR error at minimum pyruvate was +103.4%, and within a single segment of stable LPG, the LPR varied by a median of 14.4 units, driven entirely by substrate fluctuations. Pyruvate metabolism outside the LDH reaction, including transamination and carboxylation, may perturb both LPR and LPG. However, because LPG and *b* are estimated jointly by linear regression, some non-LDH-linked variation may be captured by *b* rather than LPG, potentially making LPG less susceptible than ratio-based LPR to these influences. Clinically, bedside changes in LPR may reflect pyruvate supply rather than genuine shifts in anaerobic metabolism.

The substrate delivery and LPR fidelity analyses provided converging evidence that LPR poorly reflects underlying LPG. Within segments of stable LPG, LPR fluctuations largely reflect changes in glucose and pyruvate supply (with possible hemodynamic contributions from intracranial pressure and cerebral perfusion pressure) and correspond to movement along the fitted linear lactate–pyruvate relationship. Accordingly, glucose drove LPR in opposite directions depending on the sign of *b*, illustrating the artefactual nature of these fluctuations. Between segments, LPR was a heavily attenuated proxy for LPG, partly because of a significant negative LPG–*b* coupling: as the fitted line steepens (higher LPG), *b* tends to fall, and given equation (6) (LPR = *m* + *b*/P) this opposing change partially cancels the effect of higher LPG. Although LPG was robustly and negatively associated with glucose, LPR showed a stronger negative association, reflecting summation of the true physiological signal with the substrate-dependent artefact in the majority Type P_*b*_ segments.

The clinical relevance of the LPG was established by its significant association with 6-month GOSE. Higher LPG was associated with worse outcome across a graded decline from Good recovery to Death. In contrast, the LPR–outcome association was driven primarily by the mortality group, with surviving outcome categories clustering together. The linear intercept (*b*) showed no association with outcome, in line with its role as an empirical adjustment parameter. Together, these findings position the LPG as an outcome-associated, physiologically grounded metric that the LPR only imperfectly approximates.

In the independent Uppsala cohort, several key findings were replicated: comparable segmentation output (92.7% retention, median *R*^2^ = 0.66), near-identical within-patient LPG variation (median range 11.36 vs 12.28), attenuated LPR tracking of LPG (β = 0.140 vs 0.255, consistent with a stronger negative LPG–*b* coupling), and replicated negative LPG–glucose association (β = −1.20, *p* = 0.022). The within-segment LPR–glucose association was only significant in Type P_*b*_ segments and did not reach significance in Type N_*b*_ segments (*p* = 0.090), possibly due to the smaller sample of N_*b*_ segments. The outcome association did not reach significance, most likely reflecting the smaller sample (69 vs 476 patients with outcome data) and limited power. Replication of the structural, physiological, and methodological findings across two centres nonetheless strengthens confidence in generalisability.

The in vitro experiment tested whether LPG increases when oxidative metabolism is impaired by mitochondrial dysfunction. Rotenone inhibits complex I of the electron transport chain, thereby impairing oxidative phosphorylation and altering cellular redox homeostasis; in hiPSC-derived neurons, this more than doubled LPG (from 14.2 to 32.9), supporting our interpretation of LPG as an indicator of anaerobic activity. Notably, the intercept *b* remained near zero in both conditions, suggesting that in a controlled cellular environment without the complexity of the in vivo extracellular milieu, the simplified model (L = *m*P, equation (4)) is a close approximation. Together with the in vivo glucose association, these findings provide complementary evidence that LPG captures the metabolic response to two of the primary drivers of secondary injury in TBI: substrate limitation and mitochondrial dysfunction.

This study has inherent limitations. First, our framework is retrospective: identifying linear segments requires sufficient data over time, and the accuracy of segment-level parameters depends on the duration and stability of the underlying trend. Real-time elucidation of linear trends at the bedside currently lacks feasibility with standard hourly CMD sampling. Higher-frequency or continuous monitoring may enable online LPG tracking in the future, but purpose-built algorithms for real-time changepoint detection remain to be developed. Complementary non-invasive modalities, such as hyperpolarized [1-^13^C]pyruvate magnetic resonance spectroscopy for real-time cerebral metabolic flux imaging,^23^ address overlapping metabolic questions and were not evaluated in the present CMD-focused analysis. Second, our analysis focused exclusively on segments with positive LPG (*m* > 0); while this decision was theoretically motivated by the LDH equilibrium and segments with non-positive LPG (8.6% of all segments) are likely artefactual (as discussed above), this filtering means a small proportion of monitoring time is excluded from downstream analyses. Third, extracellular lactate and pyruvate collected by CMD are influenced by cell-type-specific release and uptake, proton and substrate gradients, transporter expression and saturation, diffusion, and vascular or glymphatic clearance.^17,24,25^ CMD-derived LPG estimated across multi-hour time segments may reduce sensitivity to transient variation in these processes relative to datapoint-level LPR, but cannot eliminate systematic changes in intracellular–extracellular coupling; both LPR and LPG remain indirect proxies for aggregate local tissue redox dynamics. Quantifying these extracellular influences would require paired intracellular–extracellular measurements or tracer studies beyond the scope of this study. Fourth, the reductionist in vitro model (SM4),^26–29^ while providing evidence for the LPG–mitochondrial dysfunction link, does not wholly replicate in vivo cytoarchitecture and lacks vascular and blood-brain barrier components; it employed a single cell type and inhibitor, and further studies using alternative models of anaerobic activity would strengthen these findings. Finally, while the Uppsala cohort replicated most findings, the outcome association was not replicated, and further validation in larger cohorts is warranted.

In conclusion, this study identifies the lactate–pyruvate gradient (LPG) as a novel CMD-derived metric that provides a stable readout of anaerobic cerebral metabolism over a given data segment; it is not subject to the substrate-dependent confounding that renders the LPR an unreliable proxy. The linear lactate–pyruvate relationship is validated across two independent clinical cohorts, outcome-associated, and linked to mitochondrial dysfunction through in vitro modelling. While currently best suited for retrospective analysis, this framework establishes a foundation for developing more precise, individualised approaches to monitoring and understanding cerebral metabolism after traumatic brain injury.

## Supplemental Material

sj-pdf-1-jcb-10.1177_0271678X261485404 – Supplemental material for The microdialysis-derived lactate–pyruvate gradient indicates anaerobic activity after brain injury Supplemental material, sj-pdf-1-jcb-10.1177_0271678X261485404 for The microdialysis-derived lactate–pyruvate gradient indicates anaerobic activity after brain injury by Michael S Baker, Joshua M Heihre, Tomasz Kuliński, Claudia A Smith, Berfin Barlas, Chisomo Zimphango, Ivan Timofeev, Koby Baranes, Keri L H Carpenter, Mark Kotter, Mathew R Guilfoyle, Elham Rostami, Peter J Hutchinson and Adel Helmy in Journal of Cerebral Blood Flow & Metabolism
