# Supplementary material for: The microdialysis-derived lactate–pyruvate gradient indicates anaerobic activity after brain injury
Source: J Cereb Blood Flow Metab. 2026 Aug 26:0271678X261485404. Online ahead of print. doi: 10.1177/0271678X261485404 (PMC13558473; doi:10.1177/0271678X261485404)
Supplement: Supplementary file 1 [file sj-pdf-1-jcb-10.1177_0271678X261485404.pdf]

Article:

The microdialysis-derived lactate–pyruvate gradient indicates anaerobic activity after brain injury

Journal:

Journal of Cerebral Blood Flow & Metabolism

Authors:

Michael S Baker, Joshua M Heihre, Tomasz Kuliński, Claudia A Smith, Berfin Barlas, Chisomo Zimphango, Ivan Timofeev, Koby Baranes, Keri L H Carpenter, Mark Kotter, Mathew R Guilfoyle, Elham Rostami, Peter J Hutchinson, and Adel Helmy

Corresponding author:

Michael S Baker (Division of Neurosurgery, Department of Clinical Neurosciences, University of Cambridge, Cambridge, United Kingdom; msb74@cam.ac.uk)

Supplementary materials

SM1 Clinical management and monitoring specifications

Patients were managed according to a standardised, tiered protocol. Initial management involved sedation, paralysis, and mechanical ventilation aiming for a partial pressure of carbon dioxide between 4.5–5.0 kPa. Continuous arterial blood pressure (ABP) was monitored via a peripheral intra-arterial line, with vasopressors administered to maintain cerebral perfusion pressure (CPP) between 55 and 65 mmHg. A tiered approach was implemented to manage elevated intracranial pressure (ICP), beginning with osmotherapy and escalating to external ventricular drainage, hypothermia, barbiturate coma, and finally decompressive craniectomy if refractory.

Multimodal monitoring was established using a triple-lumen cranial access device (Technicam, Newton Abbot, UK) placed in the white matter of the non-dominant frontal lobe, unless contraindicated. This device was used to introduce sensors in the white matter for ICP

(Codman, Raynham, MA, USA), brain tissue oxygen tension ($PbtO_2$) via a Licox probe (Integra Neurosciences, Andover, UK), and cerebral microdialysis using a CMA71 (100 kDa cutoff) or CMA70 (20 kDa cutoff) catheter (CMA/M Dialysis AB, Stockholm, Sweden). The microdialysis catheter was perfused at 0.3 μL/min with a standard crystalloid solution (CNS Perfusion Fluid, CMA/M Dialysis AB). High-frequency data (50–200 Hz) for ICP, ABP, and $PbtO_2$ were captured by ICM+ software (Cambridge Enterprise/University of Cambridge, UK), which also provided bedside calculations of the pressure reactivity index (PRx). Microdialysate samples were collected hourly for bedside analysis of analyte concentrations using CMA 600, ISCUS, or ISCUSflex analysers (CMA/M Dialysis AB).

SM2 Data pre-processing details

SM2.i Initial data processing

The raw data, 56,307 observations across 619 patients, were imported using custom R scripts. Analyte measurements were discarded if the concentration fell below the lower limit of detection for the assay (glucose: 0.1 mM; lactate: 0.1 mM; pyruvate: 10.0 μM; glutamate: 1.0 μM; glycerol: 10.0 μM) or if the microdialysate sample volume was insufficient. Data from ICM+ (ICP, ABP, PRx, and $PbtO_2$) were averaged hourly and temporally aligned with the CMD data, accounting for the 17-minute transit time of the microdialysate to the collection vial. To mitigate the influence of extreme outliers, the data underwent population-level winsorisation, where values below the 0.125th percentile and above the 99.875th percentile and were set to the corresponding percentile thresholds. The resulting dataset comprised 56,250 multimodal observations from 616 patients.

SM2.ii Additional pre-processing for linear lactate–pyruvate dynamics

From each patient, we discarded the first two hours of data because the corresponding microdialysis values were presumed to be confounded by catheter insertion and probe flushing. Cerebral microdialysis concentration values falling outside the instrument's plausible ranges (lactate 0.1–12 mM, pyruvate 10–1500 µM, glucose 0.1–25 mM; based on the ISCUSflex 2017 manual) were set to null. We then excluded any observations missing lactate or pyruvate values, as these variables were central to the study. Next, we applied patient-level data sufficiency filtering, requiring each patient to have a minimum of 24 valid CMD samples (i.e., each containing values for both lactate and pyruvate) after the previous filtering steps. This threshold ensures that the segmentation pipeline has sufficient data to identify multiple temporally distinct linear trends when the data support them. Finally, pyruvate was converted from µM to mM and the LPR was computed as lactate/pyruvate. The pre-processing pipeline produced a cleaned dataset containing 50,797 total observations across 510 patients.

SM3 Uppsala cohort details

SM3.i Study population and ethics

The Uppsala cohort comprised patients with severe TBI (Glasgow Coma Scale ≤ 8) aged 18 years and over, admitted to Uppsala University Hospital, Uppsala, Sweden, between 2008 and 2020. All patients required multi-modal invasive intracranial monitoring including cerebral microdialysis. Uppsala University Hospital serves a catchment area of 1.5–2 million citizens; patients were either admitted directly or transferred from regional hospitals following initial stabilisation. Written informed consent was obtained from next-of-kin. The study was undertaken in accordance with the Declaration of Helsinki and Swedish law. Ethical approval

was granted by the Uppsala County branch of the Swedish Ethical Review Authority (#2010/138, #2010/138/1, #2010/379, #2015/224, and #2020-05462). After pre-processing (SM3.iii), the final cohort comprised 81 patients with a median age of 39 years (IQR 20–56) and was predominantly male (75.3%).

SM3.ii Clinical management and monitoring

Patient management followed Brain Trauma Foundation Guidelines, with ICP maintained below 20 mmHg and cerebral perfusion pressure above 60 mmHg. A standardised clinical management protocol focused on secondary insult prevention was implemented. Patients were mechanically ventilated and received propofol and morphine for sedation and analgesia, aiming for PaO2 ≥ 12 kPa. Patients with unstable ICP were initially hyperventilated, targeting a PaCO2 range of 4.0–4.5 kPa, and were gradually normo-ventilated as soon as ICP permitted. Systolic blood pressure was targeted above 100 mmHg. Neuromonitoring comprised an ICP monitor (external ventricular drain [HanniSet, Xtrans, Smith Medical GmbH, Glasbrunn, Germany] or intraparenchymal [Codman ICP Micro-Sensor, Codman & Shurtleff, Raynham, MA, USA]) and a CMA 71 high cut-off cerebral microdialysis catheter (100 kDa, 10 mm membrane; M Dialysis AB, Stockholm, Sweden), inserted via the same burr-hole or craniotomy in the non-dominant frontal lobe. A standard perfusion rate of 0.3 μL/min was achieved using a 106 Microdialysis pump (M Dialysis AB) with CNS perfusion fluid (M Dialysis AB; NaCl 147 mmol/L, KCl 2.7 mmol/L, $CaCl_2$ 1.2 mmol/L, $MgCl_2$ 0.85 mmol/L). Across the study period, perfusion fluid supplementation with human serum albumin followed by 3% dextran was initiated; the latter has been shown to improve inflammatory marker recovery *in vitro*. Microdialysate was analysed using ISCUSflex or CMA 600 analysers (M Dialysis AB). Functional outcome was assessed at

6–12 months post-injury using the Glasgow Outcome Scale-Extended via structured questionnaire or telephone interview.

SM3.iii Data pre-processing

Data were assembled using a custom Python script from raw data tables, executed in a secure Linux-based cluster computing environment (Uppmax Bianca). Starting from a pool of 197 patients, exclusions were applied stepwise: unknown or uncertain admission date (2 patients), very late microdialysis initiation (>14 days post-injury; 1), duplicate patient record (1), absence of bedside physiological monitoring data (13), and missing Glasgow Coma Scale in the TBI registry (3), yielding 177 patients with 9,525 microdialysis observations. This dataset was then processed through the same pre-processing pipeline applied to the Cambridge cohort (Supplementary materials SM2), which removed the first two hours of data, excluded implausible analyte concentrations, and required a minimum of 24 valid samples per patient. The pre-processing pipeline retained 8,150 observations from 81 patients. The Uppsala team adapted the published code repository for compatibility with their secure local cluster environment and added privacy-preserving steps (such as visual jitter to data outputs); these adapted scripts are available from the Uppsala team upon request.

SM4 *In vitro* experimental details

Glutamatergic neurons were generated via optimised inducible overexpression (opti-ox) of neurogenin 2 (NGN2) in human induced pluripotent stem cells (hiPSCs), in accordance with previous studies.[26–29] All cultures were maintained at 37°C in a humidified 5% CO_2 atmosphere. hiPSCs were dissociated into single cells using Accutase (Thermo Fisher Scientific, Waltham, MA, USA) and seeded onto plates coated with Geltrex (Thermo Fisher Scientific). Induction was

performed in DMEM/F12 (Thermo Fisher Scientific) supplemented with Glutamax (100X, Thermo Fisher Scientific), non-essential amino acids (100X, Thermo Fisher Scientific), 50 µM 2-mercaptoethanol (Thermo Fisher Scientific), 1% penicillin/streptomycin (Thermo Fisher Scientific), and 1 µg/mL doxycycline (Sigma-Aldrich, St. Louis, MO, USA). On Day 2, medium was switched to Neurobasal (Thermo Fisher Scientific) with B27 supplement (2%), Glutamax (100X), 50 µM 2-mercaptoethanol, 1% penicillin/streptomycin, NT3 (10 ng/mL, Peprotech, Cranbury, NJ, USA), BDNF (10 ng/mL, R&D Systems, Minneapolis, MN, USA), and 1 µg/mL doxycycline. On Day 3, neurons were seeded onto 96-well microplates coated with PDL/Geltrex at a density of 40,000 cells/cm^2 and grown in the same medium. Half media changes were performed every other day, with doxycycline withdrawn at Day 6 post-induction.

On Day 21, neurons underwent a complete media change to a test medium based on the maintenance formulation but supplemented with 5 mM glucose without exogenous lactate, pyruvate, or L-glutamine. This medium contained either vehicle or rotenone (10 nM, ≥ 95% purity; Sigma-Aldrich); rotenone stock solutions were prepared in DMSO (Sigma-Aldrich) and diluted in test medium. Samples were collected from parallel wells at timepoints between 1 and 24 hours post-treatment and analysed on an ISCUSflex analyser (M Dialysis AB).

Supplementary figures

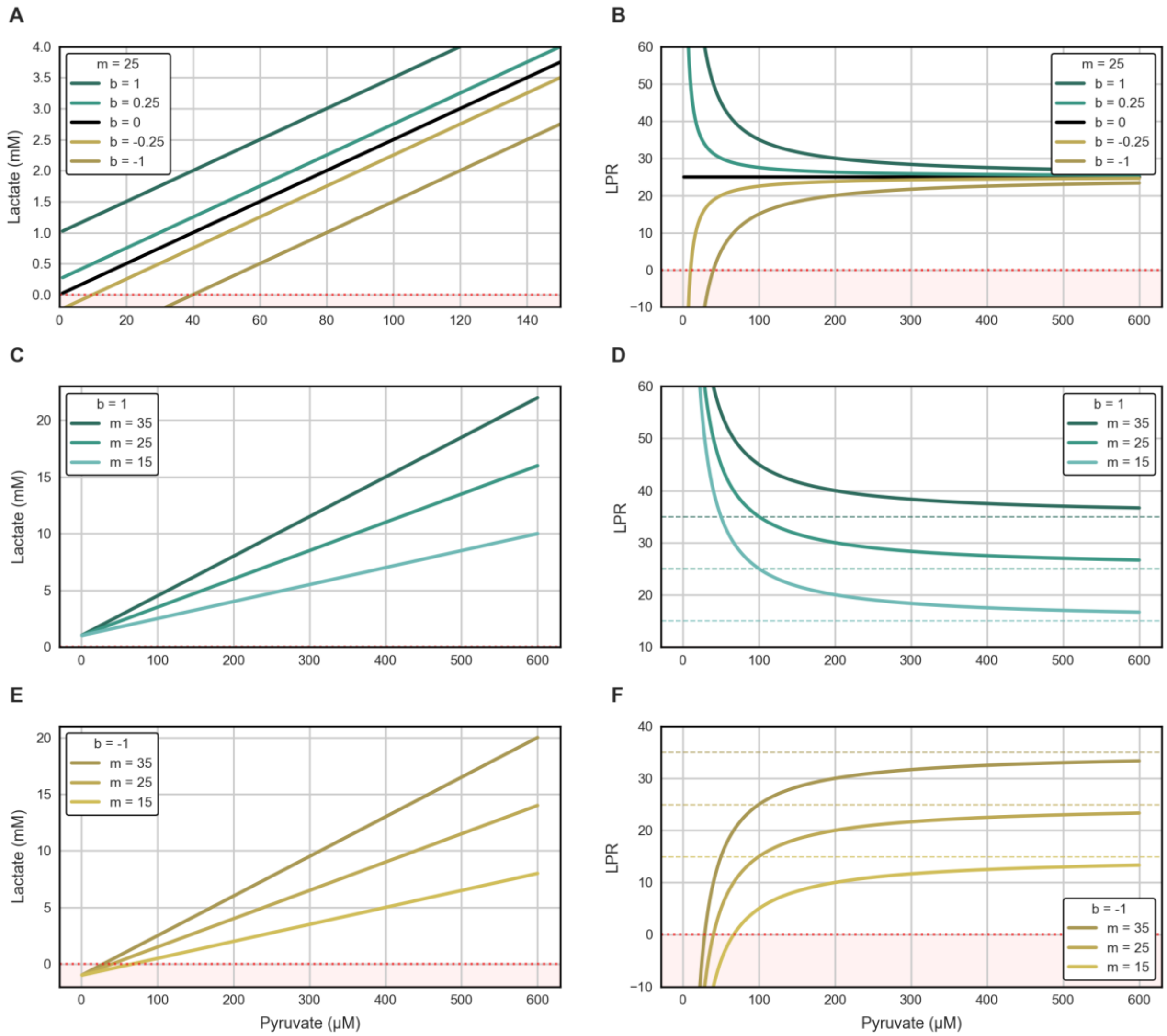

Figure S1. The linear-to-hyperbolic framework. This figure conceptually illustrates the mathematical transformation from a linear lactate–pyruvate relationship (L = *m*P + *b*) to a hyperbolic LPR–pyruvate relationship (LPR = *m* + *b*/P). (**A**–**B**) The effect of varying the intercept (*b* = 1, 0.25, 0, -0.25, -1 mM) while holding the LPG (*m*) constant at 25. (**A**) In the linear space, *b* determines the y-intercept. (**B**) In the hyperbolic space, *b* determines the direction and degree of curvature. Positive *b* values (teal) produce hyperbolae that decrease towards the LPG (*m*) asymptote, while negative *b* values (gold) produce hyperbolae that increase towards the asymptote. When *b* = 0, the LPR is constant and equal to LPG. (**C**–**D**) The effect of varying the LPG (*m* = 35, 25, 15) while holding the intercept constant at *b* = +1 mM. (**C**) In the linear space, LPG (*m*) determines the gradient (i.e., slope). (**D**) In the hyperbolic space, LPG (*m*) determines the horizontal asymptote. (**E**–**F**) The effect of varying the LPG (*m* = 35, 25, 15) while holding the intercept constant at *b* = -1 mM. Dashed lines indicate asymptotes at LPG (*m*). Red shading denotes non-physiological negative values. ***b***, linear intercept; **L**, lactate; **LPG (*m*)**, lactate–pyruvate gradient; **LPR**, lactate/pyruvate ratio; **P**, pyruvate.

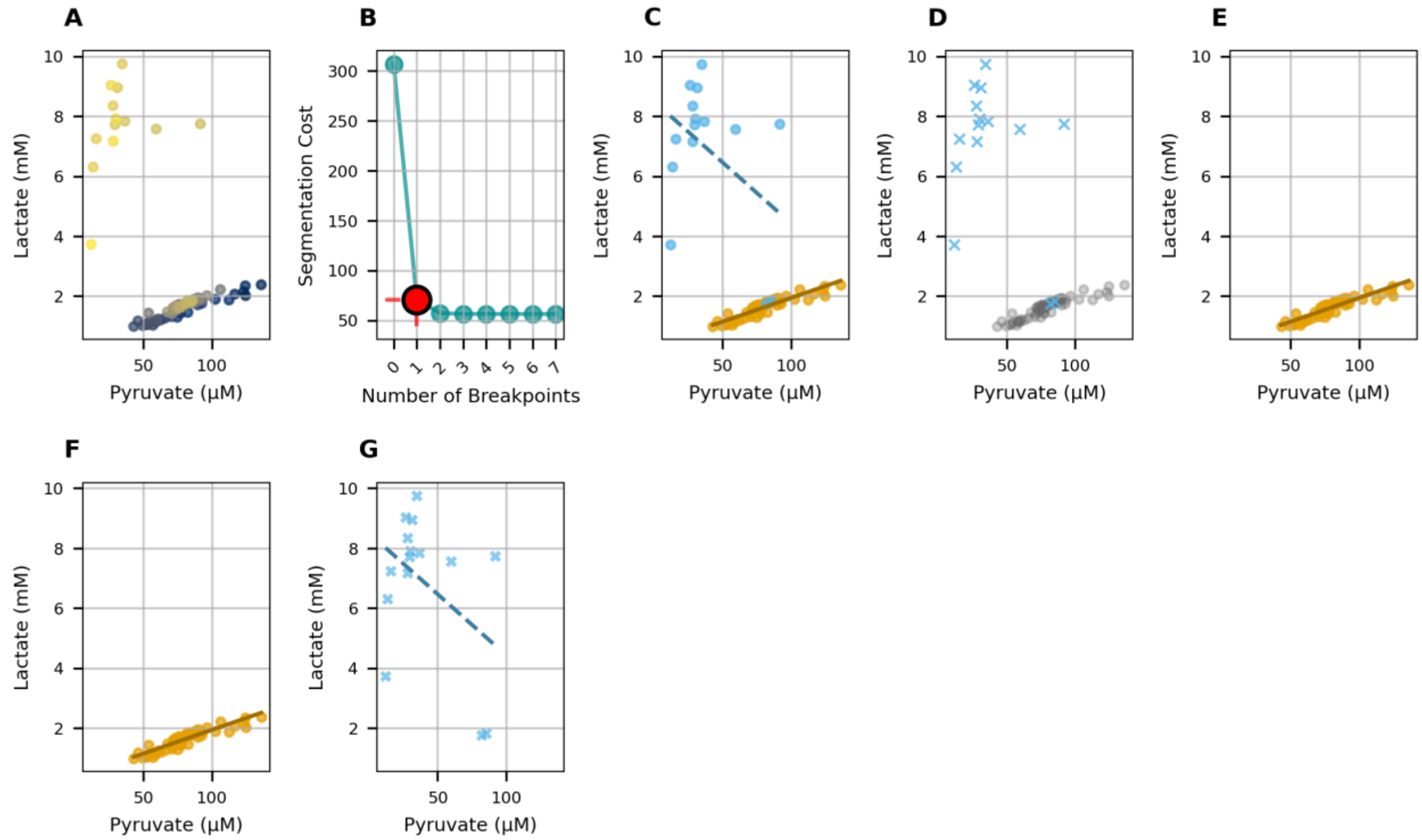

Figure S2. Linear segmentation pipeline illustration for a patient with a discarded segment. This figure demonstrates the complete pipeline process from changepoint detection through segment retention for a single patient in whom one of two identified segments was discarded due to a non-positive LPG ($m \leq 0$). (**A**) Lactate–pyruvate scatterplot for all 80 datapoints, coloured by time since injury from day 0.5 (dark blue) to day 5.2 (yellow). This panel shows the temporal structure of the data prior to segmentation, with a visually apparent cluster of high-lactate, low-pyruvate points (yellow) at the end of the monitoring period. (**B**) Cost-complexity curve from the PELT changepoint detection algorithm. A recursive bisection search across the penalty parameter space was performed to discover all unique segmentation solutions. Each teal point represents a discovered solution. The large red-and-black circle indicates the optimal solution selected by the Kneedle algorithm, which identifies the elbow point on the cost-complexity curve. For this patient, the algorithm selected 1 breakpoint (2 segments) at a segmentation cost of 70.53. (**C**) All identified segments with OLS regression lines. Two segments were identified: Segment 1 (orange, solid line; retained) and Segment 2 (blue, dashed line; discarded). (**D**) Retained datapoints from Segment 1 are shown as grey points; the discarded datapoints from Segment 2 are highlighted as blue X markers. (**E**) The retained datapoints from Segment 1 are shown with the OLS regression line. Only these data entered downstream analyses. (**F**) Segment 1 (retained): days 0.5-4.2 post-injury; 65 datapoints. OLS fit: L = 15.85P + 0.35 (Pearson r = 0.92), where L is lactate (mM) and P is pyruvate (mM). (**G**) Segment 2 (discarded): days 4.2-5.2 post-injury; 15 datapoints. OLS fit: L = -40.73P + 8.50 (Pearson r = -0.43). This segment was discarded from all downstream analyses. ***b***, linear intercept; **LPG (*m*)**, lactate–pyruvate gradient; **OLS**, ordinary least squares; **PELT**, Pruned Exact Linear Time.

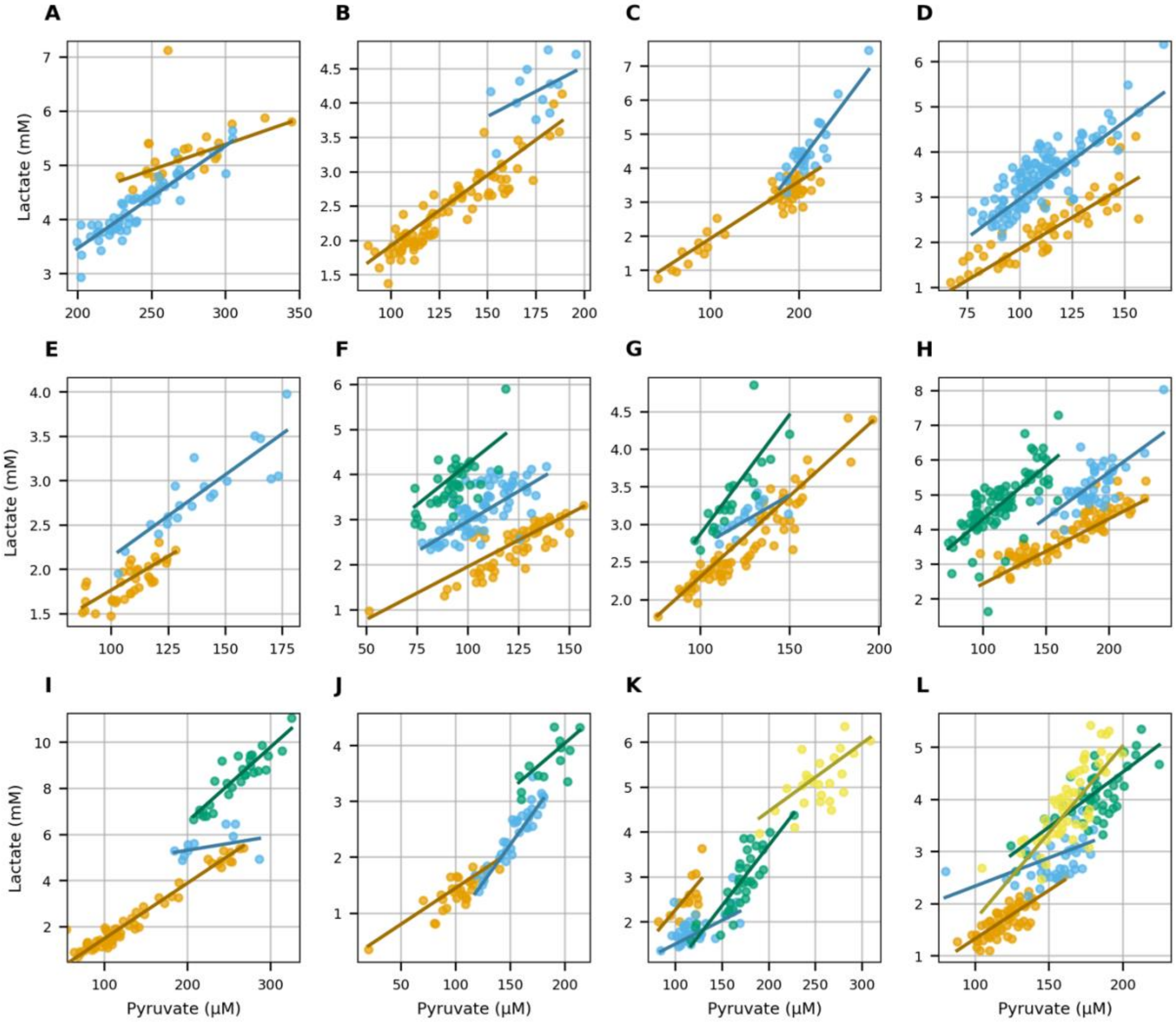

Figure S3. Lactate–pyruvate segmentation results for 12 representative patients in the Uppsala cohort. Each panel (**A**–**L**) shows one patient's segment-level scatter plots with ordinary least squares regression lines. Datapoints are coloured according to their assigned segment, which are temporally distinct and sequential; for any given patient, all datapoints belonging to 'Segment 1' occur before those in 'Segment 2', and so on. Each segment comprises a contiguous block of hourly observations bounded by changepoints. Segments are distinctly coloured in temporal order (orange first, blue second, then green). All segments shown for these patients had positive lactate–pyruvate gradients ($m > 0$).

Supplementary tables

Table S1. Segmentation pipeline input and output details

	Count (%)	Median	IQR	Range
**Input data**				
Datapoints	50,797	—	—	—
Patients	510	—	—	—
Datapoints per patient	—	88	56–135	24–279
Data span per patient (days)	—	5.2	3.4–8.0	1.2–13.6
**Segmentation results**				
Segments[a]	1,465	—	—	—
Retained segments[b]	1,339 (91.4%)	—	—	—
Datapoints across retained segments[c]	48,504 (95.5%)	—	—	—
Datapoints per retained segment[d]	—	30	20–42	8–95
Data span per retained segment (hours)[d]	—	41.4	28.3–60.4	7.9–151.4
Retained patients[e]	506 (99.2%)	—	—	—
Retained segments per retained patient	—	2	2–3	1–7

[a]Segments from the lactate–pyruvate linear segmentation pipeline.
[b]Retention requires positive LPG ($m > 0$).
[c]Datapoints in segments with $m > 0$; percentage relative to total input datapoints.
[d]Medians: cohort median (IQR) of patient-level medians.
[e]Retained patients have at least one retained segment.
IQR, interquartile range; **LPG (*m*)**, lactate–pyruvate gradient.

Table S2. LPR error relative to the LPG (*m*) across pyruvate concentrations

	P_{min} (μM)	Relative LPR error[a] at P_{min} (%)	P_{max} (μM)	Relative LPR error[a] at P_{max} (%)	LPR range
Unstratified	64 (41, 89)	66.8% (20.2%, 190.6%)	172 (139, 225)	23.3% (7.1%, 55.6%)	14.4 (9.2, 27.6)
Type P_b	61 (38, 88)	103.4% (39.1%, 254.7%)	171 (137, 225)	36.0% (16.8%, 74.5%)	16.0 (9.5, 34.7)
Type N_b	75 (51, 106)	-19.9% (-38.0%, -8.6%)	170 (140, 232)	-11.5% (-17.4%, -4.9%)	10.9 (7.5, 17.9)

Values are cohort medians (IQR) of patient-level metrics. Patient metrics: segment-weighted means (weights = segment size) of relative LPR error at segment P_{min} and P_{max}; patient medians of segment-level P_{min}, P_{max}, and LPR range. Cohort sizes: Unstratified = 506 patients, 1,339 segments; Type P_b = 476 patients, 967 segments; Type N_b = 276 patients, 372 segments.
[a]Relative LPR error = 100 × (LPR − LPG) / LPG.
b, linear intercept; **IQR**, interquartile range; **LPG (*m*)**, lactate–pyruvate gradient; **LPR**, lactate/pyruvate ratio; **N_b**, negative intercept ($b < 0$); **P_b**, positive intercept ($b > 0$); **P_{max}**, segment-maximum pyruvate; **P_{min}**, segment-minimum pyruvate.

Table S3. Comparison of linear lactate–pyruvate parameters between "Always LPR > 25" and "Never LPR > 25" cohorts

	Always LPR > 25	Never LPR > 25	Δ[a]	P-value[b]
Pre-filter % discarded ($m \leq 0$)[c]	0.0% (0.0%, 4.2%)	0.0% (0.0%, 0.0%)	0.0%	—
LPG (m)[d]	27.39 (23.82, 33.78)	14.59 (12.44, 18.20)	12.80	< 0.001
Linear intercept (b, mM)[d]	1.62 (0.58, 2.76)	0.19 (-0.15, 0.37)	1.43	< 0.001
% Type P_b	100.0% (81.3%, 100.0%)	66.3% (51.6%, 85.3%)	33.7%	< 0.001

Values are cohort median (IQR) of patient-level values. Cohort sizes: Always = 39 patients, 82 retained segments; Never = 38 patients, 92 retained segments.
[a]Δ = Always − Never.
[b]P-values from one-sided Wilcoxon tests (H1: Always > Never) with Benjamini-Hochberg correction.
[c]Pre-filter only: per-patient percent of datapoints in segments with $m \leq 0$ before filtering. All other rows use retained data ($m > 0$).
[d]Patient-level values are segment-weighted means (weights = segment size).
b, linear intercept; **IQR**, interquartile range; **LPG (*m*)**, lactate–pyruvate gradient; **LPR**, lactate/pyruvate ratio; **P_b**, positive intercept ($b > 0$).

Table S4. Segmentation pipeline input and output details for the Uppsala cohort

	Count (%)	Median	IQR	Range
**Input data**				
Datapoints	8,150	—	—	—
Patients	81	—	—	—
Datapoints per patient	—	94	54–131	24–289
Data span per patient (days)	—	5.2	3.4–7.4	1.4–13.7
**Segmentation results**				
Segments[a]	232	—	—	—
Retained segments[b]	215 (92.7%)	—	—	—
Datapoints across retained segments[c]	7,781 (95.5%)	—	—	—
Datapoints per retained segment[d]	—	30	20–45	10–110
Data span per retained segment (hours)[d]	—	37.0	28.5–55.0	14.0–129.0
Retained patients[e]	81 (100%)	—	—	—
Retained segments per retained patient	—	2	2–3	1–6

[a]Segments from the lactate–pyruvate linear segmentation pipeline.
[b]Retention requires positive LPG ($m > 0$).
[c]Datapoints in segments with $m > 0$; percentage relative to total input datapoints.
[d]Medians: cohort median (IQR) of patient-level medians.
[e]Retained patients have at least one retained segment.
IQR, interquartile range; **LPG (*m*)**, lactate–pyruvate gradient.

Table S5. LPR error relative to the LPG (*m*) across pyruvate concentrations in the Uppsala cohort

	P_{min} (µM)	Relative LPR error[a] at P_{min} (%)	P_{max} (µM)	Relative LPR error[a] at P_{max} (%)	LPR range
Unstratified	101 (78, 136)	19.1% (-0.4%, 74.9%)	196 (153, 248)	10.1% (-0.5%, 34.8%)	10.4 (7.4, 14.3)
Type P_b	94 (72, 137)	54.9% (30.6%, 122.0%)	195 (155, 258)	28.8% (14.5%, 65.7%)	10.3 (7.3, 16.5)
Type N_b	108 (86, 136)	-22.3% (-35.2%, -8.4%)	202 (160, 249)	-13.6% (-21.2%, -4.9%)	9.5 (6.1, 11.7)

Values are cohort medians (IQR) of patient-level metrics. Patient metrics: segment-weighted means (weights = segment size) of relative LPR error at segment P_{min} and P_{max}; patient medians of segment-level P_{min}, P_{max}, and LPR range. Cohort sizes: Unstratified = 81 patients, 215 segments; Type P_b = 72 patients, 132 segments; Type N_b = 61 patients, 83 segments.
[a]Relative LPR error = 100 × (LPR − LPG) / LPG.
b, linear intercept; **IQR**, interquartile range; **LPG (*m*)**, lactate–pyruvate gradient; **LPR**, lactate/pyruvate ratio; $\mathbf{N_b}$, negative intercept ($b < 0$); $\mathbf{P_b}$, positive intercept ($b > 0$); $\mathbf{P_{max}}$, segment-maximum pyruvate; $\mathbf{P_{min}}$, segment-minimum pyruvate.
